# Behavioral Determinants of Objectively Assessed Diet Quality in Obese Pregnancy

**DOI:** 10.3390/nu11071446

**Published:** 2019-06-26

**Authors:** Jasper Most, Candida J. Rebello, Abby D. Altazan, Corby K. Martin, Marshall St Amant, Leanne M. Redman

**Affiliations:** 1 Reproductive Endocrinology and Women’s Health, Pennington Biomedical Research Center, Baton Rouge, LA 70817, USA; 2 Clinical Trials Unit, Pennington Biomedical Research Center, Baton Rouge, LA 70817, USA; 3 Ingestive Behavior, Weight Management, and Health Promotion Laboratory, Pennington Biomedical Research Center, Baton Rouge, LA 70817, USA; 4 Woman’s Hospital, 100 Woman's Way, Baton Rouge, LA 70817, USA

**Keywords:** Pregnancy, obesity, diet quality, Healthy Eating Index, food cravings, mindful eating, education, race, food photography

## Abstract

Interventions to promote healthy pregnancy in women with obesity by improving diet quality have been widely unsuccessful. We hypothesized that diet quality is determined by eating behaviors, but evidence in women with obesity is lacking. We evaluated diet quality and eating behavior in 56 women with obesity (mean ± SEM, 36.7 ± 0.7 kg/m^2^, 46% White, 50% nulliparous) early in pregnancy (14.9 ± 0.1 weeks). Diet quality was objectively assessed with food photography over six days and defined by Healthy Eating Index. Eating behaviors were assessed by validated questionnaires. Women reported consuming diets high in fat (38 ± 1% of energy) and the HEI was considered “poor” on average (46.7 ± 1.3), and for 71% of women. Diet quality was independently associated with education level (*p* = 0.01), food cravings (*p* < 0.01), and awareness towards eating (*p* = 0.01). Cravings for sweets and fast foods were positively correlated with respective intakes of these foods (*p* < 0.01 and *p* = 0.04, respectively), whereas cravings for fruits and vegetables did not relate to diet intake. We provide evidence of the determinants of poor diet quality in pregnant women with obesity. Based on this observational study, strategies to improve diet quality and pregnancy outcomes are to satisfy cravings for healthy snacks and foods, and to promote awareness towards eating behaviors.

## 1. Introduction

Poor quality of maternal diet is considered one of the most significant predictors of adverse pregnancy outcomes [1,2,3,4,5] and poor infant health [5,6,7,8,9]. Both poor diet quality and adverse pregnancy outcomes are more frequent among women with obesity [10,11].

Among women with normal weight or who are overweight, improvements in maternal diet quality reduced the prevalence of adverse outcomes including excess gestational weight gain [12,13], gestational diabetes, and hypertensive disorders in mothers [2] and macrosomia in infants [14]. However, in women with obesity, successful dietary interventions are seldom reported, likely due to small effects on diet quality [14,15,16,17].

To develop more successful behavioral interventions for women with obesity requires an understanding of determinants of poor diet quality, i.e., consumption of specific food groups, mindful eating, or food cravings. Others have shown in 24 h recalls, that poor diet quality of women with obesity was determined by low intake of fruits [10], but not due to intake of other food groups. Eating behaviors such as mindful eating or food cravings associate with poor diet quality in nonpregnant subjects [18]. Women with obesity appear more likely to report indulgence in food cravings and less mindfulness towards eating, but such data has only been reported in women who were not pregnant [19]. Furthermore, the available evidence from these studies is limited by their use of self-reported diet data, varying metrics for diet quality, and lack of information on eating behaviors to understand how behaviors could be targeted to improve diet quality. To our knowledge, no study has defined the behavioral determinants of diet quality in pregnant women with obesity.

The aim of this study was to characterize diet quality in pregnant women with obesity and identify maternal eating patterns and behaviors that contribute to the quality of the diet in early pregnancy. In addition, we assessed if maternal eating attitudes and behaviors are influenced by race, education level, or nausea and vomiting. To this end, dietary intake, including diet quality and eating patterns, and eating attitudes and behaviors were simultaneously assessed between 13 and 16 weeks gestation. We hypothesized that maternal eating behavior such as food cravings and mindfulness would contribute to maternal diet quality objectively assessed by food photography.

## 2. Materials and Methods

### 2.1. Study Design

Participants were enrolled in a prospective observational study at the Pennington Biomedical Research Center to assess the determinants of gestational weight gain [20,21]. Between 13 and 16 weeks of gestation (14.9 ± 0.1 weeks), dietary intake and eating patterns were assessed over six consecutive days by a validated food photography method [22]. Eating behaviors were assessed with self-report questionnaires. The study was approved by the Pennington Biomedical Research Center Institutional Review Board and written informed consent was obtained from all participants prior to the initiation of procedures.

### 2.2. Participants

Enrolled women were pregnant, 18 to 40 years of age, with a measured BMI ≥30 kg/m^2^ at the screening visit (gestational age, <15 weeks). Obesity was defined as class I: 30≤BMI<35 kg/m^2^, class II: 35≤BMI<40 kg/m^2^, and class III: BMI≥40 kg/m^2^. Women who reported smoking, alcohol, or drug use or had pre-existing hypertension, diabetes (HbA1c ≥6.5%), psychological or eating disorders or reported medications/supplements that might affect body weight, planned termination of pregnancy, and bariatric surgery were excluded. Fasting plasma glucose was measured in a venous blood sample, collected after an overnight fast at the research center. Sociodemographic parameters including age, race, parity, family income, education, and employment were obtained from self-report questionnaires. Poverty to income ratio was determined as the ratio of individual income to poverty threshold based on family size.

### 2.3. Dietary Assessment

Dietary intake was assessed over six days with the SmartIntake^®^ smartphone application, which is a validated food photography method for adult energy intake and diet quality, as assessed by macronutrient intake (kcal from protein, fat, or carbohydrate) and intake of vitamin C, calcium, iron, sodium, or fiber [22]. Prior to analysis, routine data handling procedures were followed and days in which reported caloric intake was <60% of total daily energy expenditure measured by doubly labeled water, were excluded to improve accuracy and eliminate reporting bias by BMI [20]. Total daily energy expenditure was measured for seven days simultaneous to dietary intake [20]. When using SmartIntake^®^, participants captured images of meals or food items consumed and plate (food) waste. Participants defined the type of meal (breakfast, lunch, dinner, snacks) and every image was automatically time-stamped to allow calculation of eating patterns (first and last meal of a day, eating duration). Images were transmitted automatically in real-time via the phone application and were reviewed as a set by the participant and study staff at the end of the assessment period to document potential missing data. Dietitians determined portion sizes using validated visual comparison procedures [22], and the nutritional characteristics of each food were determined from the United States Department of Agriculture (USDA) Food and Nutrient Database for Dietary Studies 2011–2012 [23] and manufacturers’ nutrient information.

Diet quality was defined by macronutrient composition expressed relative to total energy intake (%EI), by the 2015 Healthy Eating Index (HEI) [24] and by macronutrient and micronutrient intake as compared to the intake recommendations by the Institute of Medicine [25,26]. To calculate HEI, foods and beverages were converted into the 37 USDA food pattern components [27] using the Food Patterns Equivalents Database (FPED). The FPED serves as a unique research tool to evaluate food and beverage intakes of Americans with respect to the 2015–2020 Dietary Guidelines for Americans recommendations [28]. The 37 USDA scores were converted into 12 HEI subscores measured as cup equivalents of fruit, vegetables, and dairy; ounce equivalents of grains and protein foods; teaspoon equivalents of added sugars; gram equivalents of solid fats and oils; and the number of alcoholic drinks. The HEI is a composite score of these subscores. The Healthy Eating Index 2015 ranges from 0 to 100, and HEI <51 indicates “Poor”, a score between 51 and 80 indicates “Needs Improvement” and a HEI score >80 indicates “Good”.

### 2.4. Nausea and Vomiting of Pregnancy

The Modified-Pregnancy-Unique Quantification of Emesis and Nausea Index (PUQE) questionnaire [29] is a validated instrument that assesses the severity of nausea and vomiting of pregnancy [29,30]. A PUQE Summary Score of ≤6 is considered as having no or mild symptoms of nausea and vomiting of pregnancy, a score between 7 and 12 is considered moderate and a score of ≥13 is defined as severe.

### 2.5. Eating Inventory

The Eating Inventory (EI), previously known as the Three Factor Eating Questionnaire [31,32], evaluates eating behavior in three factor domains: dietary restraint, disinhibition, and perceived hunger. A low score for dietary restraint, and high scores for disinhibition and perceived hunger, indicate less control over eating behavior and more episodic overeating.

### 2.6. Food Cravings

Food cravings were evaluated with the Food Craving Inventory (FCI), which measures the frequency of food cravings over the previous month. The FCI measures general cravings as well as cravings for high-fat foods, sweets, carbohydrates/starches, and fast food fats [33]. The version used in this study also included a scale to measure cravings for fruits and vegetables, and cravings for specific foods measured with the FCI have been found to correlate with intake of those foods [34].

### 2.7. Mindfulness

Mindfulness towards eating was assessed with the Mindful Eating Questionnaire (MEQ) which has been validated in pregnant women [35]. The MEQ explores mindfulness across five subscales, including disinhibition, awareness, external cues, emotional response, and distraction [36]. Mindful eating refers to an unbiased awareness of sensations around eating. Disinhibition measures the inability to stop eating even when full. The awareness subscale measures an individual's awareness of the sensory aspects of eating. Distraction refers to the tendency to think about other things and rush while eating. The external cues subscale refers to eating in response to environmental cues, and emotional response refers to eating in response to negative emotions. The emotional response and distraction subscales are reverse scored, and five questions on the disinhibition are reverse scored. High scores are indicative of mindful eating.

### 2.8. Statistical Analysis

The sample size was attained from available data of the prospective observational study to assess determinants of gestational weight gain (clinicaltrials.gov: NCT01954342), with energy intake measured by energy intake-balance method [37] as the primary outcome. At the given sample size (*n* = 56), the minimal detectable simple correlation for a power of 80%, at a two-sided significance level of 0.05, was *r* = 0.37. For the regression analysis, we assessed the power (1-β) of independent predictors post-hoc. Relationships between diet quality, eating patterns, and constructs of eating behavior were first evaluated by simple Pearson correlation coefficients. Significant correlations were linear; using power other than 1 did not increase the explained variability (change in R^2^ < 0.01). To test independent effects of eating behaviors on diet quality adjusted for demographic factors, we performed linear regression with HEI as the dependent variable and significant individual correlates of HEI as independent variables. Frequencies were compared using Chi-Square tests. Comparisons between African-American and White women were performed by independent Student’s *t*-test. Statistical analyses were completed using SPSS Inc. software, Version 24 for Windows (IBM Corp, Armonk, NY). Data are presented as mean ± SEM. All tests were performed with significance level α = 0.05, and findings were considered significant when *p* < α.

## 3. Results

Fifty-six women with obesity (36.7 ± 0.7 kg/m^2^, 46% White, 50% nulliparous) who collected dietary intake data that satisfied the criteria for inclusion were included in this analysis (Table 1).

### 3.1. Dietary Assessment

Participants reported consuming 3.8 ± 0.1 meals per day, of which 2.7 ± 0.05 meals were reported as main meals (breakfast, lunch, or dinner) and 1.1 ± 0.1 meals as snacks. The average reported eating duration (time between first and last photograph of any meal eaten) was 8:28 ± 0:31 h.

The mean reported energy intake was 2165 ± 43 kcal/day, which is equivalent to 84 ± 2% of energy requirements assessed by doubly labeled water during the same period. On average, participants consumed 46 ± 1% of energy as carbohydrates, 38 ± 1% as fat (41% saturated fatty acids, 35% mono-unsaturated fatty acids, 24% poly-unsaturated fatty acids), and 16 ± 1% as protein. The HEI of the diet was 46.7 ± 1.3, which is considered to reflect a “Poor” (<51) diet quality (Table 1). The HEI was positively associated with education level, but was not associated with BMI (as continuous or categorical variable; Class 1: 46.2 ± 1.9, Class 2: 45.9 ± 2.4, Class 3: 49.1 ± 2.6, *p* = 0.47), parity, race, age, income, or employment (Table 1). Breakfast consumption (*p* = 0.04) and an earlier last meal of the day (*p* < 0.01) were associated with a higher HEI.

**Figure 1:**
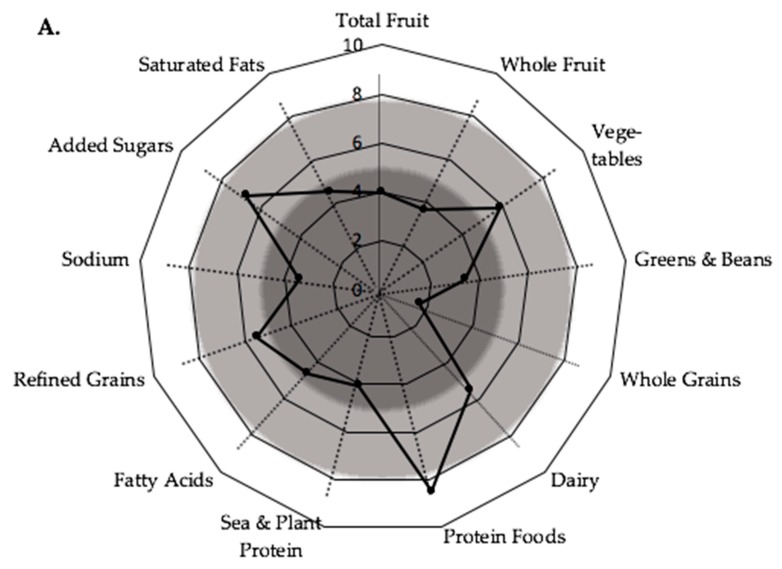

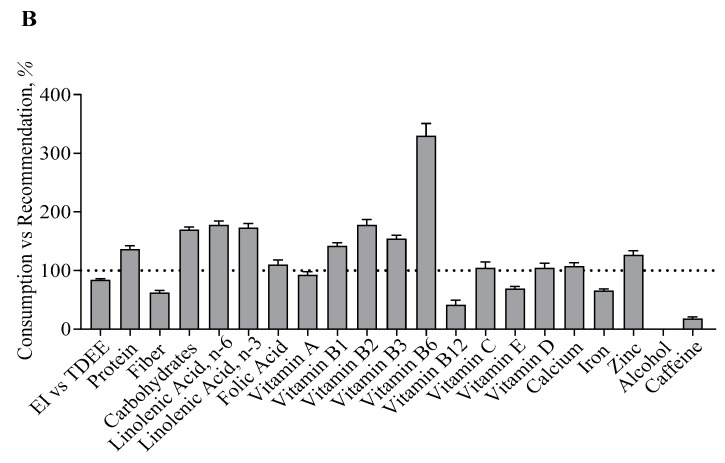
Diet Quality per Healthy Eating Index Components, and Recommendations for Intakes of Macronutrients and Micronutrients. Data are given as mean and mean ± SEM. (**A**) Values in the dark grey area indicate the respective Healthy Eating Index (HEI) food group to be consumed “poorly” (0–5), values in the light grey area as “needs improvement” (5–8) and in the white area as “sufficient” (8–10), according to the HEI guidelines [25]. HEI components scaled from 0–10 are reported as assessed and components scaled 0–5 were multiplied by two to facilitate comparability between factors. (**B**) Values are expressed as intake as compared to the minimal macronutrient and micronutrient intake recommendation by the WHO (maximum intake recommendation for alcohol and caffeine). Macronutrient and Micronutrient intake values are adjusted for the degree of underreporting per individual (−16 ± 2% vs. TDEE, adjusted intake=reported intake/reporting accuracy [“EI vs. TDEE”]).

On an individual level, 71% of women in the cohort (*n* = 40) had a HEI <51 that is “poor”, 29% had a HEI between 50 and 80 indicating that diet quality “needs improvement”, and none of the participants had a HEI >80 which is “good” diet quality (Supplemental Table S1). Scores of the individual components within the HEI are summarized in Figure 1A and Table S1. Among these, only mean intake of protein foods exceeded 80% of the HEI maximum protein score. Vegetables, dairy, refined grains, and added sugars were between 50% and 80% of the maximum scores within the respective categories, and intake of fruits (total fruits including juices, and whole fruits), greens and beans, whole-grains, seafood, and plant protein, fatty acids and saturated fats, and sodium were below 50%, and thereby “poor”.

Macronutrient and fat composition associated significantly with diet quality. HEI associated with intakes of protein (as % of total energy intake, *r* = 0.29, *p* = 0.03), but not carbohydrates. Moreover, HEI was not associated with overall fat intake, but with intakes of saturated fatty acids (*r* = −0.37, *p* = 0.005), mono-unsaturated fatty acids (*r* = 0.32, *p* = 0.02), and the ratio of unsaturated to saturated fatty acids (*r* = 0.37, *p* = 0.005). Macronutrient and micronutrient intakes as compared to the recommendations by the Institute of Medicine are presented in Figure 1B. For majority of nutrients, intakes were close to or exceeding the recommendations, but intakes of fiber (62.5 ± 3.5%), vitamin B12 (41.5 ± 8.0%), vitamin E (69.2 ± 3.8%), and iron (66.0 ± 2.9%) were particularly low.

### 3.2. Food Cravings

**Figure 2:**
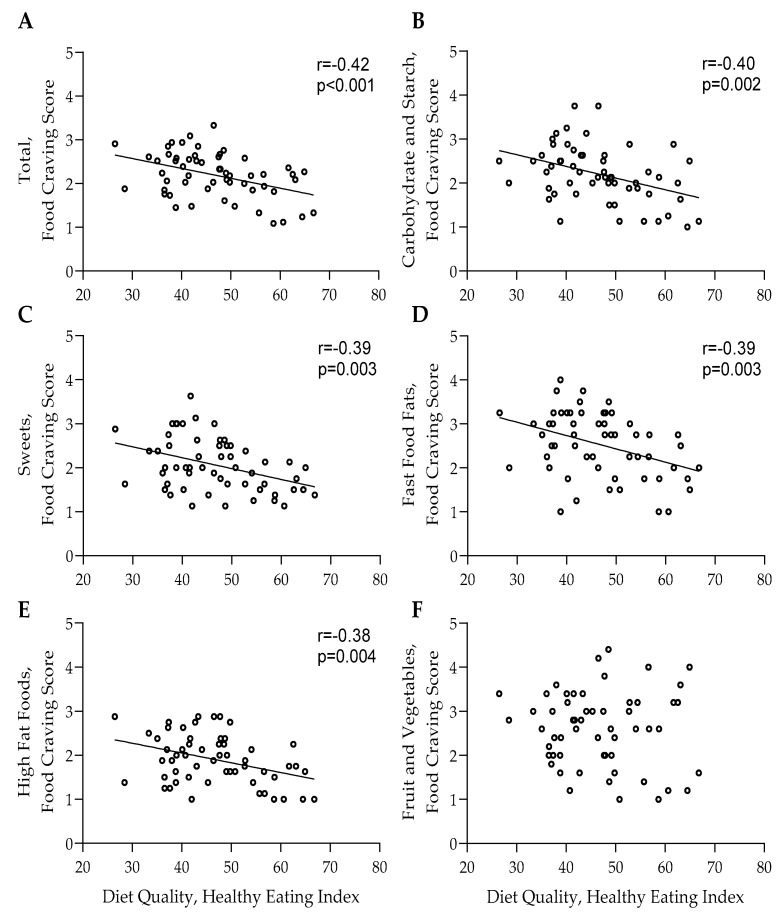
Food Cravings and Diet Quality. Food cravings scores are reported per individual in relation to their respective Healthy Eating Index. Cravings are reported as (**A**) total cravings and as cravings for (**B**) Carbohydrates and Starch, (**C**) Sweets, (**D**) Fast Food Fats, (**E**) High Fat Foods and (**F**) Fruit and Vegetables. Significant associations are indicated by linear regression.

The total food craving score was 2.19 ± 0.07, ranging from 1.09 to 3.33. The most frequent cravings were reported for “fast food fats” and “fruits and vegetables”, whereas “high-fat foods” and “sweets” were less frequently craved. In Figure 2, the associations between food cravings and diet quality were reported. Cravings for “high fat foods”, “sweets”, “carbohydrates and starches”, and “fast food fats” associated negatively with HEI, but cravings for “fruit and vegetables” did not associate with HEI.

Cravings for “sweets” correlated significantly with added sugar intake (*r* = 0.39, *p* < 0.01), but were not associated with the reported frequency of snack consumption (*p* = 0.65). Cravings for “fast food fats” were significantly associated with increased consumption of carbohydrate (*r* = 0.27, *p* = 0.04) and less consumption of fats (*r* = −0.30, *p* = 0.03). Increased cravings for both “sweets” and “fast food fats” were associated with poor consumption of whole grains, seafood, and plant proteins (“sweets”: *p* = 0.02 and *p* = 0.04, and “fast food fats”: *p* = 0.04 and *p* = 0.01, respectively). Cravings for “Fruit and Vegetables” did not correlate with intake of any HEI component.

### 3.3. Eating Inventory

The average scores from the Eating Inventory were 8.1 ± 0.6 for cognitive restraint, 5.4 ± 0.4 for disinhibition and 3.9 ± 0.3 for hunger. Fourteen (25%) women in the cohort were considered restrained eaters and one (2%) a disinhibited eater (respective subscores >12). The HEI score for diet quality was not associated cognitive restraint (*p* = 0.09), disinhibition (*p* = 0.73), or hunger (*p* = 0.43).

### 3.4. Mindful Eating

The mindful eating summary score was 2.93 ± 0.04. Overall mindfulness as assessed by the summary score was not associated with HEI (*p* = 0.22), meal/snack frequency, or timing. Subscores higher than the overall summary score indicating mindfulness towards these eating behaviors were reported for eating in response to negative emotions (“emotional eating”), inability to stop eating even when full (“disinhibition”) and the tendency to think about other things and rush while eating (“distraction”). Of the mindful eating subscores, only awareness correlated with HEI (*r* = 0.34, *p* = 0.01). Awareness associated significantly with consumption of “Greens and Beans” (*r* = 0.30, *p* = 0.02), but not with other HEI components.

### 3.5. Nausea and Vomiting and the Influence on Diet Quality

Forty-six percent of women reported no or only mild symptoms of nausea and vomiting whereas 54% experienced moderate symptoms, and none reported severe symptoms. Nausea and vomiting severity did not correlate with demographic characteristics or HEI but was associated with a lower energy intake (*p* = 0.02), skipping breakfast (*p* = 0.02), eating later in the day (*p* < 0.01), and consuming all meals during a shorter period of time (*p* < 0.01).

### 3.6. The Influence of Maternal Education on Diet Quality and Eating Behaviors

Education level was positively associated with HEI (*r* = 0.31, *p* = 0.02). Women with higher education consumed more whole grains (*p* = 0.03), greens and beans (*p* = 0.02), and less added sugars (*p* = 0.04). Women with higher education also reported eating more main meals (*r* = 0.30, *p* = 0.02), and increased awareness towards the sensory aspects of eating (*r* = 0.33, *p* = 0.01). Education level was positively associated with disinhibition scores, which indicates that higher educated women had less control over eating behavior (*r* = 0.31, *p* = 0.02). The intent to restrict food intake also was associated with education level (cognitive restraint, *r* = 0.29, *p* = 0.03) and could be in response to the tendency to overeat, as indicated by the association between disinhibition and education level.

### 3.7. Independent Effects of Awareness, Cravings, and Education

In the linear stepwise regression model with HEI as the dependent variable and significant individual correlates with HEI (*n* = 8) as independent variables, we observed that an early last meal of the day (standardized β = −0.40, *p* < 0.001, 1−β = 0.94), cravings for sweets (standardized β = −0.35, *p* = 0.004, 1−β = 0.84), and education (standardized β = 0.27, *p* = 0.02, 1−β = 0.65) were independent predictors of HEI (R^2^ = 0.38, *p* < 0.001), but not other cravings, awareness towards eating or breakfast consumption.

### 3.8. The Influence of Maternal Race on Diet Quality and Eating Behaviors

African-American women reported lower education, less income and higher parity than White women. The HEI was not significantly different between African-American and White women (AA: 45.2 ± 1.8 vs. White: 47.2 ± 1.8, *p* = 0.44). Reported energy intake as compared to energy requirements was also comparable between races. However, eating patterns and eating behaviors differed significantly by maternal race. Compared to White women, African-American women consumed their first meal later in the day (+1:28 ± 0:36 h, *p* = 0.03), and reported eating fewer snacks (African-American vs. White, −0.6 ± 0.2 snacks/day, *p* = 0.01). African-American women reported more cravings for high-fat foods and carbohydrates and starches (*p* = 0.02 and *p* = 0.03, respectively), yet less disinhibition (*p* < 0.01), indicating better control over their eating behavior compared to White women (Table 2).

## 4. Discussion

Improvements in diet quality during pregnancy can prevent poor outcomes in nonobese women [1,2,3,4,5,6,7,8,9], but dietary interventions have been largely unsuccessful in women with obesity [12,14,16,17,38,39]. We speculate that this may be explained in part by intervention approaches not being specific to the eating patterns and behaviors in women with obesity and thus achieving only small effect sizes on diet quality. Identifying maternal eating patterns that relate to diet quality in women with obesity will help inform the development of future dietary interventions with greater specificity for this group. To this end, we performed an observational cohort study and measured diet quality and eating behavior in 56 healthy women with obesity. In support of our hypothesis, diet quality in early pregnancy was poor, which was related to increased food cravings and less awareness towards eating. Nausea and vomiting severity were not associated with diet quality, but were inversely related to breakfast consumption and energy intake. Lastly, we observed that diet quality and eating behaviors differed by education and race, respectively.

Diet quality in our cohort of pregnant women with obesity was poor (HEI = 47, fat content=38%) and lower than prior reports of nonpregnancy cohorts in the US (49–64 [40,41,42,43]). Interventions in pregnant cohorts with poor diet quality may be more successful in improving maternal and infant outcomes as compared to previous lifestyle intervention studies, because the opportunity for improvement is larger. Diet quality was higher in pregnant women in Australia (HEI = 72 [38]) and the UK (fat content = 31% [15]), and diet quality improved by only 2% [12,38] to 5% [32], as assessed by healthy eating index [38], adherence score to the recommended diet [12], Glycemic Index and Glycemic Load [15].

We observed that poor diet quality is due to poor consumption of all food groups, except for protein intake, and thus appropriate interventions require approaches to target all components of the diet, e.g., Mediterranean diet, DASH diet. Nevertheless, our data also identify specific food groups and behaviors that may affect overall diet quality more than others. For example, strategies to reduce high fat intake, e.g., by reducing fast food intake, will likely reduce saturated fatty acid intake, but may also result in less refined grain consumption. Moreover, increasing fruit and vegetable consumption would likely increase dietary fiber and unsaturated fatty acid intake, thus providing a strategy to improve multiple diet quality components. Increasing fiber intake may not only improve satiety and reduce energy intake [44,45], but also improve gastrointestinal health and glucose homeostasis [46].

Food cravings increase the desire to eat and the satisfaction associated with eating [47]. In the present study, cravings for healthy foods, e.g., fruits and vegetables, were reported more frequently compared to cravings for unhealthy foods, e.g., sweets, fast food, high fat foods. Despite the frequent cravings, fruit was consumed in poor quantities according to the 2015–2020 Dietary Guidelines for Americans [29]. The inability to increase consumption of healthy foods in order to satisfy food cravings for such foods may be due to a lack of availability, perceived cost, or misperception about the health of certain foods [48]. Facilitating indulgence in cravings for healthy foods may also prevent cravings and consumption of unhealthy foods [49].

Conversely to cravings for healthy foods, cravings for foods with poor nutritional value were often indulged in and thereby contributed to poor diet quality, which is consistent with other studies [50]. Specifically, cravings for sweets were linked to intake of added sugar. This data supports and connects evidence for a causal relationship between cravings for sugar [51], consumption of sugar [52] and excess gestational weight gain. We also observed that fast food fats were frequently craved and correlated with poor diet quality, whereas high fat foods were less frequently craved. The fast food fats subscale is comprised solely of pizza, hamburgers, french fries, and potato chips, while the high fat subscale includes eight foods, including bacon, gravy, and sausage. Thus, an important factor distinguishing the two fat subscales is ease of access being that fast food fats are easier to acquire compared to foods on the high fat subscale. Hence, these data support the notion that food accessibility may influence the perception of cravings and the likelihood of eating for indulgence.

Increasing consciousness about diet choices may benefit diet quality [53,54]. We observed that awareness towards eating was associated with better diet quality. This confirms previous reports of mindfulness in pregnant women with overweight and obesity [36,55]. In addition, we show that mindfulness associates specifically with consumption of healthy foods, i.e., ‘Greens and Beans’, but not with other HEI components. Thus, whereas increasing awareness would likely increase consumption of healthy food items, no direct effect of reducing intake of unhealthy foods would be expected. To our knowledge, only one study has successfully implemented a mindfulness-based intervention in overweight, pregnant women [56,57]; however, the effect of the intervention on diet quality or pregnancy outcomes was not reported.

We hypothesized that diet quality and eating behaviors would be affected by maternal demographic characteristics such as education and race. Diet quality was poor in women with a college degree or postgraduate education (HEI = 49.2) and in women with lower education levels (HEI = 44.4, *p* = 0.06), which confirms similar findings of previous studies [58]. Women with less education were less aware of their eating behavior and had a lower intent to restrict food intake, which may reflect lacking or misguided knowledge on the risks of excess weight gain [59]. Qualitative studies in women enrolled in the Special Supplemental Nutrition Program for Women, Infants, and Children (WIC) program who generally have poor socio-economic status, report that they indeed feel uninformed about weight gain in pregnancy and that receiving more information may increase the intent to restrict food intake to optimize pregnancy outcomes [60]. Women with higher education levels had lower disinhibition scores, indicating poorer self-control. To understand the main independent effects of individual correlates with diet quality, we performed linear regression analysis with HEI as the dependent variable. We identified education, cravings for sweets and an early last meal of the day as independent predictors of diet quality. Awareness towards eating was not a significant predictor in the model, which suggests that the beneficial effect of increased awareness towards eating on diet quality is mediated by eating the last meal of the day earlier, or that education, cravings for sweets, and awareness were low/high in the same women and were thus not independent of each other. Indeed, awareness correlated significantly with both education and cravings for sweets.

We observed significant differences in eating behaviors, but not in diet quality between African-American and White women in the present study, indicating the need for different approaches to improve poor diet quality in all women. African-American women, who reported lower education levels, less income, and higher parity, consumed the first meal later in the day but not the last meal, so eating duration tended to be shorter. The severity of morning sickness was not reported to be different between African-American and White women. The observation that African-American women ate breakfast less frequently, and thus ate later in the day may have contributed to a reduced dietary intake, which we reported previously [21]. In line with these findings, African-American women also reported better control over their eating behavior and reported less snacking despite more cravings.

The strength of this study is the use of food photography. Food photography methods are more accurate in measuring portion sizes and the composition of meals since these are analyzed in an objective manner [22]. Furthermore, the food photography method is performed in real time and is not prone to recall bias as with instruments reliant on recall, yet possibly to “attention bias” for the reporting of snacks [20]. With respect to energy intake, implementation of data quality criteria in the analytical stages minimizes the degree of under-reporting to less than 20% compared to doubly labeled water and eliminates increased reporting bias typically observed with higher energy intake [20]. The major weakness of this study is the lack of prepregnancy data. Thereby, we were unable to determine whether poor diet quality and associated eating behaviors can be targeted prior to pregnancy. Further, the observation period (six days) was relatively short as compared to other instruments such as a food frequency questionnaire which recalls intake over a period of 1 to 12 months. Eating behaviors were assessed by questionnaires and are prone to self-reporting bias. Importantly, all questionnaires were previously validated in pregnancy. In addition, micronutrient, but not macronutrient, intakes may be higher than reported due to prenatal vitamin supplement intake. Lastly, this study did not enroll women without obesity, and therefore we were unable to compare diet quality and eating behaviors between women with normal weight, overweight, or obesity. Early pregnancy diabetes was excluded by HbA1c, but no glucose tolerance test was performed to determine gestational diabetes.

## 5. Conclusions

The main behavioral determinants of poor diet quality in pregnant women with obesity include the low consumption of fruits and vegetables, the indulgence into cravings for sweets, and eating late in the day. Future intervention strategies developed with a specificity for improving diet quality are therefore to increase the availability of fruits and vegetables rather than sweets or fast foods, which may facilitate indulgence in the observed cravings for healthy foods, and to reduce late-night snacking. In addition, women with lower education levels may benefit from educational interventions on the risks of overeating, whereas women with higher education may benefit from strategies to increase self-control. Future studies are required to show whether strategies to change those behaviors can be successfully implemented and improve pregnancy outcomes in women with obesity.

## Figures and Tables

**Table 1:** Subject Characteristics.

	All, *n* = 56	Association with Diet Quality, Healthy Eating Index
	Mean ± SEM	*r*, *p*
Race, *n* (African-American, White, Others)	25, 26, 5	0.11, 0.43
Gestational Age, weeks	11.3 ± 0.3	-
Maternal Age, years	28.3 ± 0.6	0.19, 0.16
Body weight, kg	97.8 ± 2.2	-
Body mass index, kg/m^2^	36.7 ± 0.7	0.01, 0.96
Obesity Class, *n* (1, 2, 3)	23, 21, 12	-
Fasting Plasma Glucose, mmol/L (range)	4.9 ± 0.1 (4.2, 6.1)	0.17, 0.20
Education, *n* (1, 2, 3)	6, 39, 11	0.31, 0.02
Employment, *n* (1, 2, 3, 4)	1, 14, 9, 32	0.18, 0.18
Household Income *	3.6 ± 0.3	0.14, 0.30
Parity, *n* (0, 1, ≥2)	28, 16, 12	−0.10, 0.46
Healthy Eating Index	46.7 ± 1.3	-

Education is categorized into High School (1), college (2), postgraduate work (3). Employment into medically disabled (1), unemployed (2), part-time employment (3), and full-time employment (4). * Household income was computed as a percent of federal poverty line (“poverty” < 1.0) according to family size. Parity is defined as number of previous pregnancies of viable infant >20 weeks gestation.

**Table 2:** Eating Behavior Assessment.

	African-American, *n* = 26	White, *n* = 25	*p* for Race	Range	High Values Indicative of
**Food Cravings**					
High Fat	2.12 ± 0.11	1.76 ± 0.10	0.02	1–5	More Craving
Sweets	2.12 ± 0.12	2.04 ± 0.12	0.64	1–5	More Craving
Carbohydrates and Starch	2.42 ± 0.11	2.03 ± 0.13	0.03	1–5	More Craving
Fast Food Fats	2.65 ± 0.12	2.47 ± 0.18	0.41	1–5	More Craving
Fruit and Vegetables	2.79 ± 0.16	2.45 ± 0.18	0.16	1–5	More Craving
Total Score	2.36 ± 0.09	2.08 ± 0.11	0.06	1–5	More Craving
**Mindful Eating**					
Awareness	2.58 ± 0.12	2.53 ± 0.10	0.77	1–4	Mindfulness
Distraction	3.24 ± 0.10	2.95 ± 0.13	0.09	1–4	Mindfulness
Disinhibition	3.34 ± 0.08	3.00 ± 0.10	0.01	1–4	Mindfulness
Emotional Cues	3.54 ± 0.09	3.32 ± 0.10	0.11	1–4	Mindfulness
External	2.29 ± 0.11	2.69 ± 0.10	0.01	1–4	Mindfulness
Summary Score	2.99 ± 0.06	2.89 ± 0.06	0.24	1–4	Mindfulness
**Eating Inventory**					
Cognitive Restraint	8.08 ± 1.04	7.69 ± 0.91	0.78	0–21	Greater Control
Disinhibition	4.20 ± 0.47	6.69 ± 0.66	0.004	0–16	Less Control
Hunger	3.52 ± 0.42	4.50 ± 0.40	0.10	0–14	Less Control
**PUQE**					
Summary Score	6.48 ± 0.43	5.54 ± 0.4	0.12	3–15	Severe Symptoms

PUQE, Modified-Pregnancy-Unique Quantification of Emesis and Nausea Index.

## Supplementary Materials

The following are available online at https://www.mdpi.com/2072-6643/11/7/1446/s1, Table S1: HEI components.
