# Supplementary material for: Behavioral Determinants of Objectively Assessed Diet Quality in Obese Pregnancy
Source: Nutrients. 2019 Jun 26;11(7):1446. doi: 10.3390/nu11071446 (PMC6682916; doi:10.3390/nu11071446)
Supplement: Supplementary file 1 [file nutrients-11-01446-s001.pdf]

Behavioral determinants of objectively assessed diet quality in obese pregnancy

Jasper Most[1], Candida J Rebello[2], Abby D Altazan[1], Corby K Martin[3], Marshall St Amant[4, 5], Leanne M Redman[1]

[1] Reproductive Endocrinology and Women's Health Laboratory, Pennington Biomedical Research Center, 6400 Perkins Road, Baton Rouge, LA 70808

[2] Clinical Trials Unit, Pennington Biomedical Research Center, 6400 Perkins Road, Baton Rouge, LA 70808

[3] Ingestive Behavior, Weight Management, and Health Promotion Laboratory, Pennington Biomedical Research Center, 6400 Perkins Road, Baton Rouge, LA 70808

[4] LSU Health Sciences Center, 433 Bolivar St, New Orleans, LA 70112

[5] Woman's Hospital, 100 Woman's Way, Baton Rouge, LA 70817

Supplemental Data

Supplemental Table 1. HEI components

Supplemental Table 1. HEI components

Category	All, n=56	Average Score	Distribution Good, Needs Improvement, Poor, in %	Range	Standard for Maximum Score
Healthy Eating Index	46.7±1.3	Poor	0, 29, 71	0-100	
Total Fruit	2.0±0.2	Poor	16, 11, 73	0-5	≥ 0.8 cup eq/1000 kcal
Whole Fruit	1.8±0.2	Poor	14, 13, 73	0-5	≥ 0.4 cup eq/1000 kcal
Vegetables	2.9±0.2	Needs Improvement	14, 50, 36	0-5	≥ 1.1 cup eq/1000 kcal
Greens and Beans	1.7±0.2	Poor	9, 25, 66	0-5	≥ 0.2 cup eq/1000 kcal
Whole Grains	1.6±0.3	Poor	2, 7, 91	0-10	≥ 1.5 oz/1000 kcal
Dairy	5.5±0.3	Needs Improvement	14, 45, 41	0-10	≥ 1.3 oz/1000 kcal
Total Protein Foods	4.3±0.1	Good	68, 30, 2	0-5	≥ 2.5 oz/1000 kcal
Seafood and Plant Proteins	2.0±0.2	Poor	13, 23, 64	0-5	≥ 0.8 oz/1000 kcal
Fatty Acids	4.6±0.4	Poor	13, 27, 61	0-10	(PUFAs+MUFAs)/SFAs ≥ 2.5
Refined Grains	5.5±0.4	Needs Improvement	20, 38, 43	0-10	≤ 1.8 oz/1000 kcal
Sodium	3.4±0.3	Poor	5, 16, 79	0-10	≤ 1.1 g/1000 kcal
Added Sugars	6.8±0.4	Needs Improvement	43, 27, 30	0-10	≤ 6.5% of energy
Saturated Fats	4.5±0.3	Poor	5, 34, 61	0-10	≤ 8% of energy

Data are given as mean±sem. The lowest and highest observed values as well as the potential ranges are provided for each food group category.
